# Prediction of cardiovascular adverse events in newly diagnosed multiple myeloma: Development and validation of a risk score prognostic model

**DOI:** 10.3389/fonc.2023.1043869

**Published:** 2023-03-21

**Authors:** Shuai Yuan, Jie-Yi Zhou, Ben-Zhao Yang, Zhong-Lei Xie, Ting-Jun Zhu, Hui-Xian Hu, Rong Li

**Affiliations:** ^1^ Shanghai Institute of Cardiovascular Disease, Zhongshan Hospital, Fudan University, Shanghai, China; ^2^ Department of Nuclear Radiation Injury Protection and Treatment Department, Naval Medical Center, Naval Medical University, Shanghai, China; ^3^ Department of Cardiology, Naval Medical Center, Naval Medical University, Shanghai, China; ^4^ Department of Hematology, Affiliated Jinhua Hospital, Zhejiang University School of Medicine, Jinhua, Zhejiang, China; ^5^ Department of Hematology, The Myeloma and Lymphoma Center, Shanghai Changzheng Hospital, Naval Medical University, Shanghai, China

**Keywords:** multiple myeloma, cardiovascular adverse events, prediction model, treatment plan, cardiovascular protection

## Abstract

**Background:**

Multiple myeloma (MM) is the second most common hematological malignancy, and the treatments markedly elevate the survival rate of the patients in recent years. However, the prevalence of cardiovascular adverse events (CVAEs) in MM had been increasing recently. CVAEs in MM patients are an important problem that we should focus on. Clinical tools for prognostication and risk-stratification are needed.

**Patients and methods:**

This is a retrospective study that included patients who were newly diagnosed with multiple myeloma (NDMM) in Shanghai Changzheng Hospital and Affiliated Jinhua Hospital, Zhejiang University School of Medicine from June 2018 to July 2020. A total of 253 patients from two medical centers were divided into training cohort and validation cohort randomly. Univariable analysis of the baseline factors was performed using CVAEs endpoints. Multivariable analysis identified three factors for a prognostic model that was validated in internal validation cohorts.

**Results:**

Factors independently associated with CVAEs in NDMM were as follows: age>61 years old, high level of baseline office blood pressure, and left ventricular hypertrophy (LVH). Age contributed 2 points, and the other two factors contributed 1 point to a prognostic model. The model distinguished the patients into three groups: 3–4 points, high risk; 2 points, intermediate risk; 0–1 point, low risk. These groups had significant difference in CVAEs during follow-up days in both training cohort (*p*<0.0001) and validation cohort (*p*=0.0018). In addition, the model had good calibration. The C-indexes for the prediction of overall survival of CVAEs in the training and validation cohorts were 0.73 (95% CI, 0.67–0.79) and 0.66 (95% CI, 0.51–0.81), respectively. The areas under the receiver operating characteristic curve (AUROCs) of the 1-year CVAEs probability in the training and validation cohorts were 0.738 and 0.673, respectively. The AUROCs of the 2-year CVAE probability in the training and validation cohorts were 0.722 and 0.742, respectively. The decision-curve analysis indicated that the prediction model provided greater net benefit than the default strategies of providing assessment or not providing assessment for all patients.

**Conclusion:**

A prognostic risk prediction model for predicting CVAEs risk of NDMM patients was developed and internally validated. Patients at increased risk of CVAEs can be identified at treatment initiation and be more focused on cardiovascular protection in the treatment plan.

## Introduction

Multiple myeloma (MM) is a malignant disease with abnormal proliferation of clonal plasma cells, which leads to a series of target organ dysfunction and clinical manifestations (1). MM accounted for 1%–1.8% of all malignant tumors, and it is the second most common malignancy of the blood system (2). The global epidemic of MM still continues in recent years. The incidence of MM from 1990 to 2016 have increased by 126% globally (3). More than 155,688 people were diagnosed with MM worldwide in 2019 (4). A total of 100,000 people a year die of MM on average (5). In China, it was estimated that the incidence of MM increased significantly from 2006 to 2016 as well. In addition, the mortality was increased from 2006 to 2014 but remained stable from 2014 to 2016, which may be due to the maturity of hematopoietic stem cell transplantation (HSCT) and the application of new drugs such as proteasome inhibitors (PI) and immune modulators (IMiDs) (6). With the development of these “novel agents” in the past decade, the progression-free survival (PFS) and overall survival (OS) of MM patients have prolonged significantly. MM has gradually evolved into a kind of chronic relapsing disease (5). Therefore, it is important to have a good management on comorbidities and therapy-related toxicities to improve clinical outcomes and enhance the quality of life in these patients.

Cardiovascular adverse events (CVAEs) are common in MM. It includes increased risk of venous thromboembolic events (VTEs), arterial thromboembolic events (ATEs), hypertension, arrhythmia, ischemic heart disease, pulmonary hypertension, and heart failure (HF) (7). An observation study showed that the risk of any cardiac event, arrhythmia, HF, and cardiomyopathy was significantly higher for MM patients exposed to three or more types of therapy than for non-MM patients who were age and gender matched (8). Two large population-based study also demonstrated that the risk of vascular complications including VTE and ATE was significantly increased (9, 10). The reason of high incidence of CVAEs in MM can be broadly divided into two aspects. First of all, the median age of diagnosis in MM is 69 years, and 63% of MM patients were more than 65 years at the time of first diagnosis (5), which means a high baseline incidence of traditional cardiovascular risk factors in MM patients. It was estimated that approximately 66% of patients had cardiovascular disease at baseline (11). On the other hand, the risk of CVAEs was closely associated with the progression of MM. Chronic renal insufficiency and amyloidosis related to MM potentially lead to the high incidence of CVAEs (12, 13). As a consequence, it is important to assess the cardiovascular risk at baseline and control the cardiovascular complications in MM patients. Early recognition and making individual treatment strategies may improve outcomes in patients with MM.

Risk stratification can help identify different levels of risk patients upfront and enable informed therapeutic decision making. Although several risk factors have been associated with CVAEs in MM patients in previous studies (14), a validated clinical tool that could be used for risk adapted treatment approaches is lacking. Here, we explored the potential risk factors associated with CVAEs after chemotherapy for MM patients and reported a three-factor prognostic model that is intuitive and easy to use for predicting the CVAEs in MM patients.

## Methods

### Patients

We conducted a retrospective study on 253 consecutive patients with NDMM treated in Shanghai Changzheng Hospital of China and Affiliated Jinhua Hospital, Zhejiang University School of Medicine of China between June 2018 to June 2020. A total of 201 patients were from Shanghai Changzheng Hospital of China, and 52 patients were from the Affiliated Jinhua Hospital, Zhejiang University School of Medicine of China. The International Myeloma Working Group (IMWG) criteria was used to assess the diagnosis and treatment response. Patients with diseases such as Waldenström macroglobulinemia, lymphoma, plasma cell leukemia, systemic light chain amyloidosis (AL amyloidosis), and MM patients who previously had received chemotherapy were excluded. The clinical information was collected retrospectively by reviewing the patients’ medical records. Several baseline variables were selected in this study, including age, sex, body mass index (BMI), body surface area (BSA), Durie–Salmon (D-S) stage, International Staging System (ISS) stage, smoking and alcohol consumption history, history of hypertension, coronary heart disease, diabetes, and stroke, baseline office blood pressure, left ventricular ejection fraction (LVEF), left ventricular mass index (LVMI), first-line therapy regimens, C-reactive protein (CRP), hemoglobin, brain natriuretic peptide (BNP), albumin, uric acid, creatinine, glomerular filtration rate (GFR), β2 microglobulin (β2-MG), and high-risk cytogenetic abnormalities (high-risk CA) [high-risk CA includes t(4; 14), gain (1q), del (17p), t (14; 16), and t (14; 20)]. The primary outcome was CVAE. The Common Terminology Criteria for Adverse Events (CTCAE) Version 5.0 was assessed for the occurrence of CVAEs. Overall survival (OS) of CVAEs was calculated from the beginning of first-line chemotherapy until the date of confirming CVAEs or the last date that the patient was known to be free of CVAEs. This study has been approved by the Ethics Committee of Shanghai Changzheng Hospital and followed the principles of the Declaration of Helsinki.

### Statistical analysis

The cohort was divided into two groups randomly: a training cohort comprising 75% of the original group and a validation cohort consisting of the remaining 25%. The training cohort used to establish the Risk Score System. In the training cohort, the baseline clinical features mentioned above were assessed to identify predictors of CVAEs. The baseline continuous variables of normal distribution were represented by mean and standard deviation. Non-normal continuous variables were represented by median and quartile range (IQR); categorical variables were expressed as counts and percentages. Univariate analysis of potential risk factors for CVAEs was performed using the Cox proportional hazards regression model. Variables in the univariate analysis with *p*< 0.10 were chosen for multivariate Cox proportional hazard regression to identify the independent prognostic factors. Based on the results of the multivariate Cox regression analyses, the Risk Score System to predict the risk of CVAEs for NDMM patients was formulated.

We validated the prognostic performance of the model by identifying and calibrating measurements in the training, validation, and entire cohort. Concordance index (C-index) was used to assess the predictive power of the model. The calibration of the prediction model was performed by a visual calibration plot comparing the predicted and actual probability of CVAEs. The outcome discrimination is most often assessed by calculating the area under the curve (AUC) of the receiver operating characteristic (ROC) curve. The closer the AUC value is to 1, the better discrimination capacity the prediction model has. We also adopted bootstrapping methods (1,000 bootstrap resamples) for internal validation to quantify the extent of model overfitting and optimism.

Additionally, we have printed decision curve analysis (DCA) curves to assess the clinical utility of this prediction model. All data were analyzed using R software (version 4.0.3, R Foundation), and p <0.05 was considered statistically significant.

## Results

### Rate of CVAEs: All types of CVAEs

During a median follow-up of 12.5 months, 74 patients (29.2%) experienced CVAEs in the entire cohort; 41 patients (17.0%) experienced Grade 3 or greater CVAEs (CTCAE≥3). The subtypes of CVAEs occurring during treatments are listed in **Table 1**. Heart failure (35.1%), arrhythmias (33.8%), and hypertension (18.9%) were the most commonly represented CVAEs. Premature beats (23.0%) are the major type of arrhythmias. In addition, heart failure (53.7%) is also the most common type in Grade≥3 CVAEs.

**Table 1:** Lists of all types of CVAE.

CVAEs		Patients n=74	CVAEs	
			Grade 1–2 (n=33)	Grade≥3 (n=41)
Heart failure		26(35.1)	4(12.1)	22(53.7)
Arrhythmia	Premature beats	17(23.0)	11(33.3)	6(14.6)
	Atrial fibrillation	5(0.07)	2(6.1)	3(7.3)
	PSVT	3(0.04)	0	3(7.3)
Hypertension		14(18.9)	10(30.3)	4(9.8)
ACS		3(0.04)	0	3(7.3)
Pulmonary hypertension		2(0.03)	2(6.1)	0
Pericardial effusion		4(0.05)	4(12.1)	0

CVAEs, cardiovascular adverse events; ACS, acute coronary syndrome; PSVT, paroxysmal supraventricular tachycardia.

### Clinical characteristics of the training and validation cohorts

The baseline clinical characteristics of the training and validation cohorts are presented in **Table 2**. A total of 253 patients were included in our study. The median age was 63.0 years [interquartile range (IQR), 56.0–68.0 years]. There were 151 men (59.7%) and 102 (40.3%) women. Everyone was treated with proteasome inhibitors (PI), and 124 patients (46.2%) accepted the treatment of immunomodulator drugs (IMiDs), including lenalidomide and thalidomide. A total of 34 patients (13.4%) accepted treatment of anthracycline, including doxorubicin and doxorubicin hydrochloride liposome. A total of 253 patients from two medical centers were divided into training cohort and validation cohort randomly. The randomization was performed through a computerized random numbers procedure by R software and conducted independently of the study investigators. The training cohort comprised 190 patients (75% of the original group), and the validation cohort consisted of 63 patients (25% of the original group). There were no significant differences between the two groups of patients in age, sex, MM type, D-S stage, ISS stage, high-risk CA rate, smoking and alcohol consumption history, history of hypertension, coronary heart disease, diabetes mellitus, and stroke, baseline CRP, hemoglobin, BNP, creatine, albumin, uric acid, β2-MG, GFR, baseline high level of office blood pressure rate, LVEF, LVMI, first-line therapy regimens, and combined CV-related drugs.

**Table 2:** The baseline characteristics of the train and validation cohorts.

Factors	Subgroup	Overall (n=253)	Train cohort(n=190)	Validation cohort(n=63)	*p*-value
Age,years,median(IQR)		63.0 [56.0, 68.0]	63.0 [57.0, 68.0]	62.0 [55.0, 67.0]	0.25
Sex, n (%)	Female	102 (40.3)	79 (41.6)	23 (36.5)	0.57
	Male	151 (59.7)	111 (58.4)	40 (63.5)	
Type, n (%)	Non-secretory	12 (4.7)	11 (5.8)	1 (1.6)	0.16
	Light-chain	44 (17.4)	28 (14.7)	16 (25.4)	
	IgA	60 (23.7)	44 (23.2)	16 (25.4)	
	IgD	13 (5.1)	10 (5.3)	3 (4.8)	
	IgE	1 (0.4)	1 (0.5)	0 (0.0)	
	IgG	120 (47.4)	95 (50.0)	25 (39.7)	
	IgM	3 (1.2)	1 (0.5)	2 (3.2)	
D-S, n (%)	I	10(4.0)	9(4.7)	1(1.6)	0.18
	II	28(11.1)	18(9.5)	10(15.9)	
	III	215(85.0)	163(85.8)	52(82.5)	
ISS, n (%)	I	53 (20.9)	40 (21.1)	13 (20.6)	0.95
	II	96 (37.9)	73 (38.4)	23 (36.5)	
	III	104 (41.1)	77 (40.5)	27 (42.9)	
High-risk CA, n (%)	No	151 (59.7)	117 (61.6)	34 (54.0)	0.36
	Yes	102 (40.3)	73 (38.4)	29 (46.0)	
Smoke, n (%)	No	172 (68.0)	130 (68.4)	42 (66.7)	0.92
	Yes	81 (32.0)	60 (31.6)	21 (33.3)	
Alcohol consumption, n (%)	No	210 (83.0)	156 (82.1)	54 (85.7)	0.64
	Yes	43 (17.0)	34 (17.9)	9 (14.3)	
BSA, m^2^, median (IQR)		1.7 [1.6, 1.8]	1.7 [1.6, 1.8]	1.7 [1.6, 1.8]	0.59
Hypertension, n (%)	No	156 (61.7)	114 (60.0)	42 (66.7)	0.43
	Yes	97 (38.3)	76 (40.0)	21 (33.3)	
Coronary heart disease, n (%)	No	226 (89.3)	170 (89.5)	56 (88.9)	1
	Yes	27 (10.7)	20 (10.5)	7 (11.1)	
Atrial fibrillation, n (%)	No	247 (97.6)	186 (97.9)	61 (96.8)	1
	Yes	6 (2.4)	4 (2.1)	2 (3.2)	
Heart failure	No	251 (99.2)	189 (99.5)	62 (98.4)	1
	Yes	2 (0.8)	1 (0.5)	1 (1.6)	
Diabetes mellitus, n (%)	No	232 (91.7)	175 (92.1)	57 (90.5)	0.89
	Yes	21 (8.3)	15 (7.9)	6 (9.5)	
Stroke, n (%)	No	228 (90.1)	172 (90.5)	56 (88.9)	0.89
	Yes	25 (9.9)	18 (9.5)	7 (11.1)	
CRP, mg/L, median (IQR)		3.1 [1.0, 7.1]	3.0 [1.0, 6.3]	4.0 [1.2, 8.9]	0.16
Hb, g/L, median (IQR)		98.0 [80.0, 118.0]	98.0 [76.5, 119.0]	103.0 [84.5, 113.5]	0.44
BNP, pg/ml, median (IQR)		134.0 [54.7, 554.0]	145.0 [56.2, 545.5]	120.0 [42.8, 589.5]	0.57
Cr, μmol/L, median (IQR)		75.0 [62.0, 103.0]	75.0 [61.2, 102.5]	76.0 [63.0, 109.0]	0.33
Alb, g/L, median (IQR)		34.6 (7.0)	34.6 (6.9)	34.6 (7.4)	0.95
Uric acid, μmol/L, median (IQR)		377.0 [306.0, 474.0]	375.0 [306.0, 471.5]	401.0 [313.5, 486.5]	0.37
β2-MG, mg/L, median (IQR)		4.3 [3.0, 7.0]	4.1 [3.0, 7.5]	4.6 [2.9, 6.7]	0.7
GFR, ml/min, median (IQR)		69.5 [49.7, 97.8]	68.9 [49.7, 99.3]	70.5 [49.1, 93.7]	0.53
Baseline HBP, n (%)	No	197 (77.9)	147 (77.4)	50 (79.4)	0.88
	Yes	56 (22.1)	43 (22.6)	13 (20.6)	
LVEF, %, median (IQR)		65.0 [62.0, 68.0]	65.0 [62.0, 68.0]	65.0 [62.0, 67.0]	0.42
LVMI, g/m^2^, median (IQR)		96.6 [82.1, 110.8]	96.6 [82.1, 111.1]	98.9 [82.7, 108.8]	0.88
First therapy regimens contains					
iMiDs, n (%)	No	129 (51.0)	99 (52.1)	30 (47.6)	0.64
	Yes	124 (49.0)	91 (47.9)	33 (52.4)	
Anthracycline, n (%)	No	219 (86.6)	167 (87.9)	52 (82.5)	0.39
	Yes	34 (13.4)	23 (12.1)	11 (17.5)	
Combined CV-related drugs					
Aspirin	No	222 (87.7)	165 (86.8)	57 (90.5)	0.59
	Yes	31 (12.3)	25 (13.2)	6 (9.5)	
ACEI/ARB	No	216 (85.4)	157 (82.6)	59 (93.7)	0.05
	Yes	37 (14.6)	33 (17.4)	4 (6.3)	
Beta-blockers	No	234 (92.5)	177 (93.2)	57 (90.5)	0.67
	Yes	19 (7.5)	13 (6.8)	6 (9.5)	
CCB	No	202 (79.8)	149 (78.4)	53 (84.1)	0.43
	Yes	51 (20.2)	41 (21.6)	10 (15.9)	
Statin	No	227 (89.7)	170 (89.5)	57 (90.5)	1
	Yes	26 (10.3)	20 (10.5)	6 (9.5)	
Diuretics	No	242 (95.7)	182 (95.8)	60 (95.2)	1
	Yes	11 (4.3)	8 (4.2)	3 (4.8)	

D-S, Durie–Salmon staging system; ISS, International Staging System; high-risk CA, high-risk cytogenetic abnormalities; CRP, C-reactive protein; Hb, hemoglobin; Cr, creatine; β2-MG, β2-microglobulin; IMiDs, immunomodulatory drugs; CCB, calcium channel blockers; CV, cardiovascular.

### The risk factors of CVAEs

Patients in the study cohort were followed up for a median of 12.5 months. CVAE occurred in 74 patients (29.2%). To screen the better variables related to CVAEs, we had transformed continuous variables into categorical variables by drawing 1- and 2-year survival ROC curves and the cutoff value in the training cohort. We had transformed the continuous variables that have clinical significance and the area under the curve (AUC) >0.5(**Supplementary Figure**). Finally, age was divided into two groups by 61 years old, β2-MG was divided into two groups by 3.71 mg/L, and uric acid was divided into two groups by 439 mmol/L. Univariate and multivariate analyses were performed on baseline indicators for the training set (**Table 3**). In the univariate analysis, age group, history of smoking, history of atrial fibrillation, baseline high level of office blood pressure (defined as blood pressure≥140/90mmHg), LVEF, left ventricular hypertrophy (LVH) (defined as >125 g/m^2^ in men and >120 g/m^2^ in women), β2MG group were significantly associated with CVAEs. Multivariate analyses were performed using the significant risk factors determined in the univariate analysis, and age group, baseline high level of office blood pressure, and LVH were revealed as significant independent factors for CVAEs. Grade 3 or greater CVAEs (CTCAE≥3) received more attention in clinical situation; univariate and multivariate analyses were also performed on baseline indicators for the entire cohort (**Table 4**). In the univariate analysis, history of smoking, diagnosis of ISS stage above stage III, baseline high level of office blood pressure (defined as blood pressure≥140/90mmHg), LVH (defined as >125 g/m^2^ in men and >120g/m^2^ in women), β2MG group, and GFR were significantly associated with Grade 3 or greater CVAEs. Multivariate analyses were performed using the significant risk factors determined in the univariate analysis; history of smoking and LVH were revealed as significant independent factors for Grade 3 or greater CVAEs.

**Table 3:** Univariate and multivariate Cox analyses for OS of CVAEs in patients with NDMM in training cohort.

Variable	Univariate	*p*	Multivariate	*p*
	HR (95% CI for HR)		HR	
Age group	6.967 (2.993-16.220)	<0.001	4.935(2.063-11.804)	<0.001*
Smoke	1.967 (1.166-3.318)	0.011	1.632(0.940-2.834)	0.115
Atrial fibrillation	4.601 (1.656-12.780)	0.003	2.150(0.716-6.460)	0.073
Baseline HBP	3.072 (1.818-5.190)	<0.001	1.795(1.031-3.125)	0.018*
LVEF	0.944 (0.892-0.999)	0.049	0.961(0.905-1.019)	0.176
LVH	2.598 (1.373-4.917)	0.003	2.208(1.110-4.391)	0.022*
β2MG group	1.969 (1.093-3.547)	0.024	1.093(0.567-2.110)	0.889
Diuretic	2.963 (1.267-6.928)	0.0122	1.746(0.696-4.381)	0.235

Baseline HBP, baseline high level of office blood pressure; LVEF, left ventricular ejection fraction; LVH, left ventricular hypertrophy; β2-MG, β2-microglobulin. *P<0.05.

**Table 4:** Univariate and multivariate Cox analyses for OS of Grade 3–5 CVAEs in patients with NDMM in entire cohort.

Variable	Univariate	*p*	Multivariate	*p*
	HR (95% CI for HR)		HR	
Smoke	2.799 (1.498-5.232)	0.001*	2.3256 (1.2033-4.495)	0.012*
DM	2.138 (0.894-5.110)	0.088	1.0267(0.3645-2.892)	0.960
Stroke	2.210 (0.964-5.066)	0.061	1.6827 (0.6534-4.333)	0.281
ISS above III	2.085 (1.118-3.890)	0.021*	1.0138(0.4228-0.4228)	0.976
Baseline HBP	2.272 (1.164-4.434)	0.016*	1.8977(0.9525-3.781)	0.069
LVH	3.410 (1.703-6.827)	<0.001*	2.8347(1.3313-6.036)	0.007*
β2MG group	2.497 (1.222-5.101)	0.012*	1.3768(0.5368-3.532)	0.506
GFR	0.989 (0.980-0.998)	0.015*	1.0029(0.9864-1.008)	0.601

DM, diabetes mellitus; ISS above III, ISS stage above III; Baseline HBP, baseline high level of office blood pressure; LVH, left ventricular hypertrophy; β2-MG, β2-microglobulin; GFR, glomerular filtration rate. *P<0.05.

### Construction and validation of the risk score system

**Figure 1:**
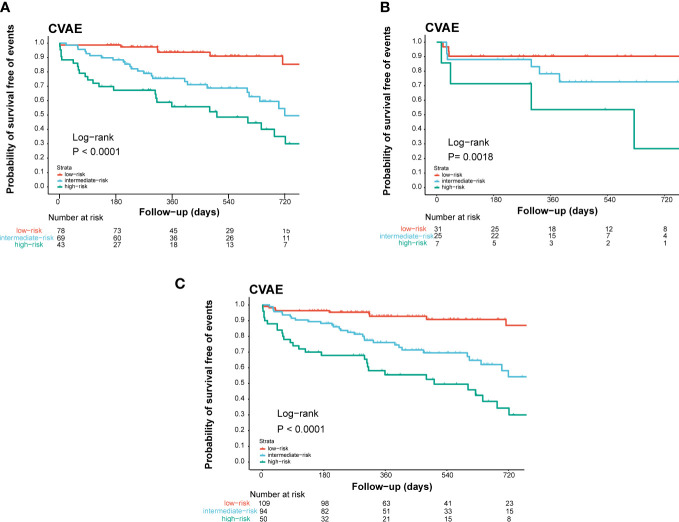
Validation of the prognostic index of CVAEs occurrence in NDMM patients. Kaplan–Meier estimates of CVAE-free survival in **(A)** the training cohort, **(B)** the internal validation cohort, and **(C)** the combined cohort validation cohort. High, high-risk group; Int, intermediate-risk group; Low, low-risk group.

Based on these results, age >61 years old, baseline high level of office blood pressure, and LVH were identified as risk factors of CVAEs. Regression coefficients for each co-variate were rounded to the nearest integer to derive weights to develop the risk score prognostic model (**Supplementary Table**). Age >61 years old contributed 2 points, and the other two factors contributed 1 point to a prognostic model. We developed a risk score system to predict the occurrence of CVAE in 1 and 2 years. On the basis of the separation of CVAEs occurrence in the training set by the cumulative number of risk factors (**Figure 1**), we distinguished three prognostic risk groups: 3–4 points, high risk; 2 points, intermediate risk; and 0–1 point, low risk. In both training and validation cohort, the intermediate- and high-risk groups had significantly inferior OS of CVAEs when compared with the low-risk group (**Table 5**). In the training cohort, the intermediate- and high-risk groups had significantly increased risk in CVAEs when compared with the low-risk group (*p*<0.0001). In the validation cohort, the intermediate- and high-risk groups had significantly increased risk in CVAEs when compared with the low-risk group as well (*p*=0.0018). When combining all cohorts, the 1-year CVAEs risk for low-, intermediate-, and high-risk groups were 6.4%, 22.3%, and 42.0%, and the 2-year CVAEs risk were 8.3%, 31.9%, and 56.0%, respectively (*p*<0.001).

**Table 5:** Survival of the clinical risk groups stratified by the three-factor model.

Risk Group	Three-Factor Score	No. Patients	HR^a^(95%CI)	*p*
Training cohort		190		
Low	0-1	78	1	–
Int	2	69	4.965 [2.034, 12.123]	<0.01
High	3-4	43	10.822 [4.474, 26.175]	<0.01
Validation cohort		63		
Low	0-1	31	1	–
Int	2	25	3.018 [0.776, 11.734]	0.11
High	3-4	7	9.624 [2.271, 40.790]	<0.01

HR, hazard ratio; a, Comparison of the low-risk group with other risk groups within the same cohort.

**Figure 2:**
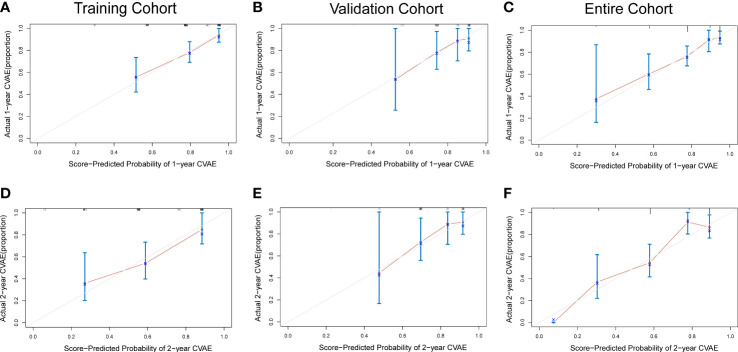
Calibration curves for predicting 1- and 2-year CVAE-free survival in the **(A, D)** training cohort, **(B, E)** validation cohort, and **(C, F)** entire cohort.

**Figure 3:**
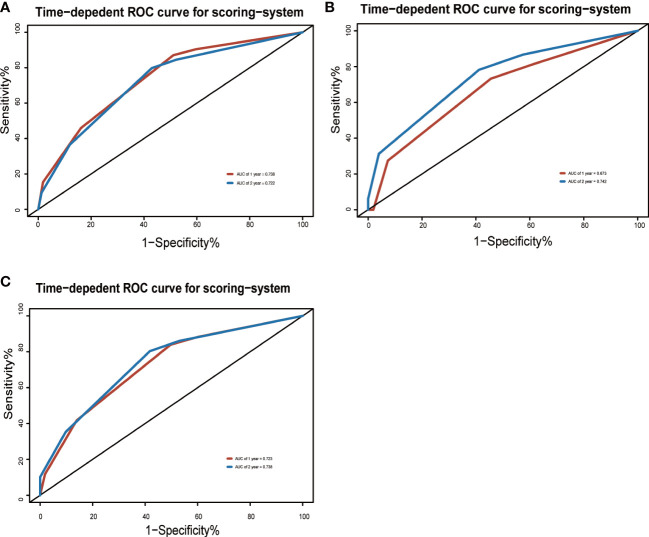
Area under the ROC curves of the three-factor risk score system in the **(A)** training cohort, **(B)** validation cohort, and **(C)** entire cohort.

To adjust optimism, bootstrapping approach was used for internal validation. The discrimination power of the risk score system was evaluated by the C-index values and ROC curves. The C-indexes for the prediction of OS of CVAEs in the training cohorts were 0.73 (95% CI, 0.67–0.79), and the calibration plots showed good agreement between the predicted OS of CVAEs and the observed OS of CVAEs rate (**Figures 2A–D**). The C-indexes for the prediction of OS of CVAEs in the validation and entire cohorts were 0.66 (95% CI, 0.51–0.81) and 0.71 (95% CI, 0.65–0.77), respectively. The calibration plots also showed good agreement between predictions and observations in both validation cohort (**Figures 2B–E**) and entire cohort (**Figures 2C–F**). The areas under ROC curves (AUROCs) of the 1-year CVAEs probability in the training and validation cohorts were 0.738 and 0.673, respectively. The AUROCs of the 2-year CVAEs probability in the training and validation cohorts were 0.722 and 0.742, respectively (**Figure 3**).

### Clinical value of the risk score system

**Figure 4:**
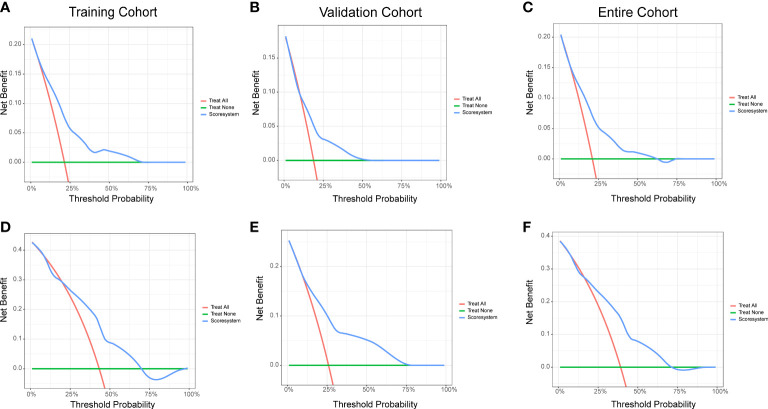
Decision curve analysis of the risk score system for the CVAE-free survival prediction of NDMM patients. **(A)** One-year survival in the training cohort. **(B)** One-year survival in the validation cohort. **(C)** One-year survival in the entire cohort. **(D)** Two-year survival in the training cohort. **(E)** Two-year survival in the validation cohort. **(F)** Two-year survival in the entire cohort.

The decision-curve analysis (DCA) is a better approach than AUROC in evaluating prognostic strategies, and it had been widely used in recent years (15). The 1- and 2-year DCA curves for the risk score system in training, validation, and entire cohorts are presented in **Figure 4**. The DCA curves indicated that the prediction model provided greater net benefit than the default strategies of providing or not providing assessment for all patients.

## Discussion

We developed a prognostic scoring system highly predictive of the risk of CVAEs in MM patients. Age >61 years old, high level of baseline office blood pressure, and LVH were independently associated with the occurrence of CVAEs. In addition, LVH was also independently associated with the occurrence of severe CVAEs (CTCAE≥3). The three-factor scoring system stratified the risk of CVAEs in our cohort into three groups: high-, intermediate-, and low-risk groups. The occurrence of CVAEs showed significant difference among the three groups of patients during a mean follow-up of 12.5 months. The C-indexes of this scoring system for predicting the occurrence of CVAEs were 0.73 (95% CI, 0.67–0.79) in the training set, 0.66 (95% CI, 0.51–0.81) in the validation set, and 0.71 (95% CI, 0.65–0.77) in entire cohort. The C-indexes and ROC curves demonstrated that this scoring system showed excellent individually predictive effects in predicting the occurrence of CVAEs of patients with NDMM in the training, validation, or entire cohort.

There are several advantages in this prognostic model. First of all, the three-factor model is practical and easy to implement in general practice because it is built on parameters that are the most common indications in clinical testing. Second, MM is a group of very heterogeneous disorders, and a variety of treatment plans were used in MM patients in real-world situation. The patients in the study cohort were based on real treatment plan of MM and were followed up to determine the occurrence of CVAEs. The prognostic model constructed by this kind of data may reflect the real condition on the occurrence of CVAEs in MM. Third, several cardiovascular risk assessment protocols have been proposed in cancer patients undergoing cardio-toxic treatment in previous studies (16–18). However, few of them could apply to MM patients. In recent years, a prospective study that investigated the relationship between Carfilzomib (CFZ) therapy in MM and CVAEs has established a risk score for CVAEs in MM patients (19). However, this risk score system is inapplicable in predicting the occurrence of CVAEs in MM, which have a range of different treatment plans. To our knowledge, it is the first prognostic model applied to predict the occurrence of CVAEs for MM in real treatment regimens.

In our prognostic model, age >61 years old, high level of baseline office blood pressure, and LVH were revealed as significant independent factors for CVAEs. Age contributed 2 points, and the other two factors contributed 1 point to a prognostic model. In contrast, several classic clinical factors, such as the history of cardiovascular disease, BNP, creatinine, and GFR, did not have consistent or independent prognostic value. Several factors have related to this result. First, only two independent medical centers were incorporated in our study, and a limited number of subjects were included. These traditional factors were not enough to make a difference during our follow-up time. Second, the NDMM patients who have the history of cardiovascular disease might have received more attention on cardiovascular protection and have less cardiovascular toxicity regimens in the beginning of the therapy. Lastly, age, LVH, and baseline office blood pressure were also closely related to cardiac dysfunction. Until now, age is the best predictor of cardiovascular disease (CVDs) (20, 21). The risk of cardiovascular disease increases with age (22). LVH was one of echocardiographic characteristics of the left ventricle that has been studied in recent years. Most LVH are associated with chronic stress, volume overload, and ischemic disease from a population standpoint (23). The ability of LVM to predict CVD outcomes has been demonstrated in earlier studies. After adjusting the other baseline characteristics, baseline LVM was considered to have a significant predictive ability in the incidence of CVD, CVD-related death, and all-cause mortality in the Framingham Heart Study (24). Recent studies showed a continuous relationship between LVM and CVD events as well. It was reported that every 39 g/m^2^ increase in LVM was associated with a 40% increase in cardiovascular disease risk (25). This continuous relationship has also been validated in a prospective study (26). It is needed to study further about the relationship between LVH and CVAEs after MM treatment. Abnormal baseline office blood pressure was considered as undiagnosed, untreated, or uncontrolled hypertension. Hypertension is the leading risk factor for CVD (27). High blood pressure that was undiagnosed or inadequately controlled with medication accounts for a significant portion (28). It means that we should pay attention to blood pressure monitoring in the treatment of NDMM. The underlying mechanisms between abnormal baseline office blood pressure and CVAEs might be blood vessel damage and stiffening. A study showed that Carfilzomib could increase coronary perfusion pressure, resting vasoconstricting tone, and the spasmogenic effect of different agents (29). Further studies are warranted to verify and expand on the relationship between blood pressure and MM treatment.

However, our study still has some limitations that undermine its generalizability. First, it is a retrospective study, and data bias exists. Second, our risk score system was developed and validated using data from two medical centers, and the lack of external validation may limit its wide application. Third, a relatively small number of patients were enrolled in our study, and the median follow-up time was only 12.5 months. The longer-term CVAEs cannot be accurately assessed. Fourth, although there were no significant differences in baseline treatment regimens, there were still differences in treatment regimens among patients. Some drugs have presented cardiovascular toxicity definitely in previous studies such as Doxorubicin (DOX) (30, 31). However, this study did not show a clear association between DOX and CVAEs. The reason may be that the number of DOX patients was not enough to reflect the statistical difference, and DOX may not develop CVAEs with statistical difference during our follow-up period. Thus, the results in our study need further prospective studies and external validation by other research centers to ensure its clinical applicability.

## Conclusion

In conclusion, we developed a prognostic risk prediction model for predicting CVAE risk of NDMM patients. The internal validation showed good performance in predicting 1- and 2-year CVAEs. Patients at increased risk of CVAEs can be identified at treatment initiation and be more focused on cardiovascular protection in treatment plan.

## Data availability statement

The raw data supporting the conclusions of this article will be made available by the authors, without undue reservation.

## Ethics statement

Written informed consent was obtained from the individual(s) for the publication of any potentially identifiable images or data included in this article.

## Author contributions

SY, J-YZ, B-ZY, H-XH and RL designed the research and drafted the manuscript. T-JZ and Z-LX presided over the enrollment and exclusion of patients and followed up the patients and collected the data. Z-LX checked the data. SY and J-YZ statistically analyzed the data. RL supervised the conduct of the study and revised the manuscript. All authors contributed to the article and approved the submitted version.
