# Supplementary figures and images for: Prediction of cardiovascular adverse events in newly diagnosed multiple myeloma: Development and validation of a risk score prognostic model
Source: Front Oncol. 2023 Mar 21;13:1043869. doi: 10.3389/fonc.2023.1043869 (PMC10070977; doi:10.3389/fonc.2023.1043869)

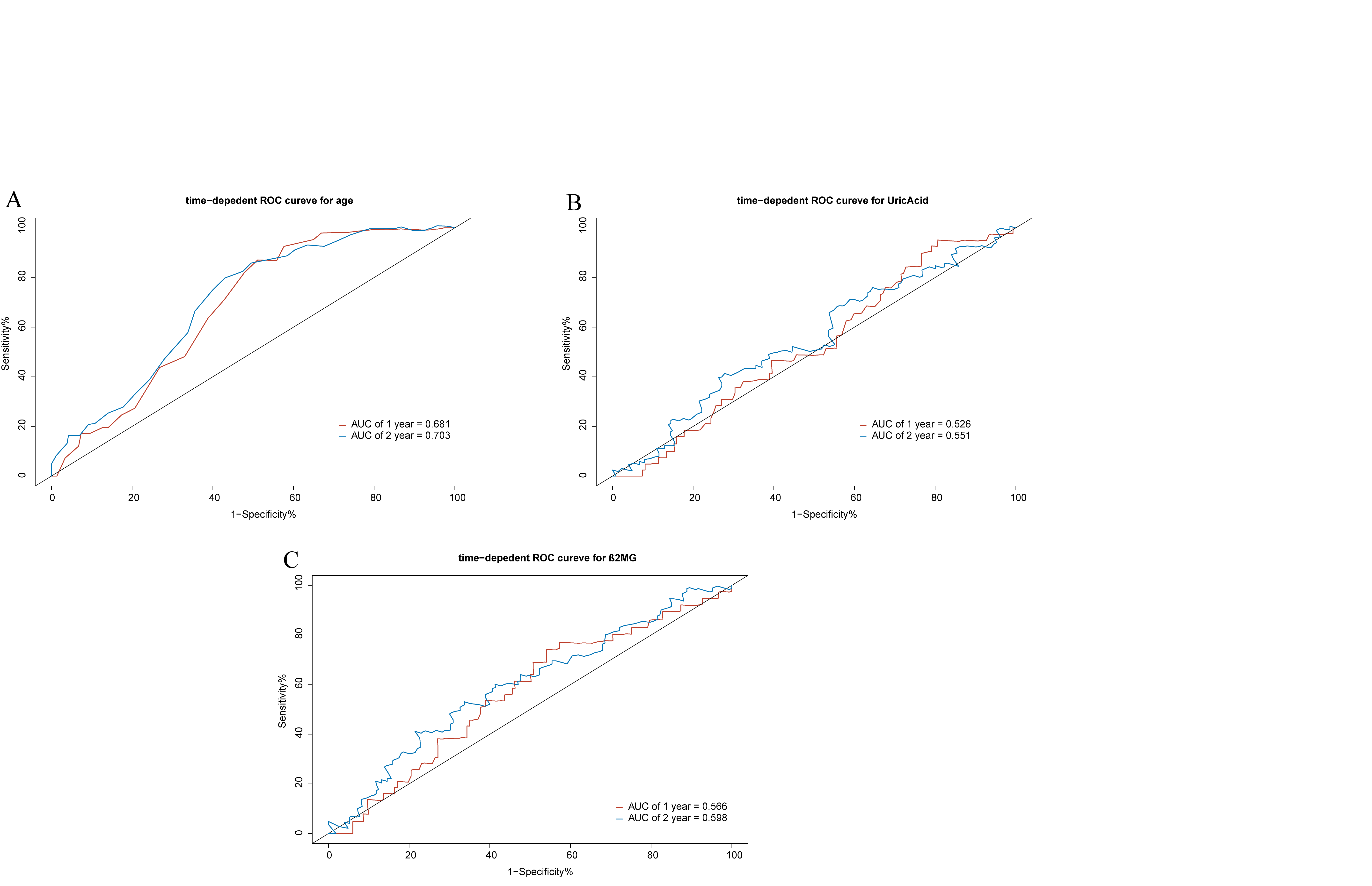

Supplement: Supplementary file 2 [file Image_1.tif]
